# Use of microfasteners to produce damage tolerant composite structures

**DOI:** 10.1098/rsta.2015.0277

**Published:** 2016-07-13

**Authors:** Ivana K. Partridge, Stephen R. Hallett

**Affiliations:** Department of Aeronautical Engineering, University of Bristol, Queen’s Building, University Walk, Bristol BS8 1TR, UK

**Keywords:** composite, Z-pin, tuft, delamination resistance, crack bridging models, cohesive zone

## Abstract

The paper concerns the mechanical performance of continuous fibre/thermosetting polymer matrix composites reinforced in the through-thickness direction with fibrous or metallic rods or threads in order to mitigate against low delamination resistance. Specific illustrations of the effects of microfasteners in reducing delamination crack growth are made for Z-pinned and tufted composites. Response to loading in such ‘structured materials’ is subject to multiple parameters defining their in-plane and out-of-plane properties. Single microfastener mechanical tests are well suited to establish the crack bridging laws under a range of loading modes, from simple delamination crack opening to shear, and provide the basis for predicting the corresponding response of microfastener arrays, within a given material environment. The fundamental experiments on microfasteners can be used to derive analytical expressions to describe the crack bridging behaviour in a general sense, to cover all possible loadings. These expressions can be built into cohesive element constitutive laws in a finite-element framework for modelling the effects of microfastener arrays on the out-of-plane mechanical response of reinforced structural elements, including the effects of known manufacturing imperfections. Such predictive behaviour can then be used to assess structural integrity under complex loading, as part of the component design process.

This article is part of the themed issue ‘Multiscale modelling of the structural integrity of composite materials’.

## 1. Introduction

The class of composite materials made by combining high-performance continuous carbon fibres with a stiff thermosetting resin matrix has long been the material of choice for advanced composite structures in the aerospace industry, because of its high specific stiffness. The high specific strength in any fibre-dominated direction is also an attraction, but the brittleness of the solid matrix resin surrounding the fibres has always been a concern. Significant damage can propagate within a layered composite laminate by cracking of the matrix resin between the plies. This traditional low-delamination resistance can be tackled successfully by the insertion of rigid microfasteners in the through-thickness direction, at least as far as resistance to crack growth is concerned. This much has been established for the case of thin rigid composite rods (Z-pins) inserted into a prepreg stack prior to cure [1–3] and for thin threads stitched or tufted into dry fibre preforms prior to resin infusion and cure [4,5]. Such through-thickness reinforcement has been demonstrated to deliver significant increases in delamination resistance under mode I-dominated loading modes in quasi-static as well as impact and fatigue conditions and has resulted in a number of current industrial applications in the aerospace and automotive sectors.

The balance of out-of-plane and in-plane properties of such structured materials dependent not only on the parameters of the *z*-direction reinforcement, such as the material of the microfastener, the areal density of reinforcement, any deviation from the intended insertion path or insertion depth, but also on the stacking sequence of the fibrous bed and the thickness of the composite. Parametric studies are therefore best carried out via multi-scale modelling, based on experimental determination of the crack bridging laws from a single microfastener placed in the relevant structural environment [6,7].

**Figure 1.**
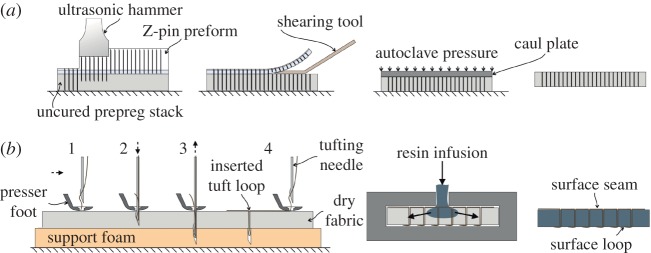
Through-thickness reinforcement of composites: schematic of process of (*a*) Z-pinning and (*b*) tufting. (Online version in colour.)

Figure 1 shows a schematic of the current one-sided insertion industrial processes by which the microfasteners are placed into (i) a resin-preimpregnated uncured composite stack (Z-pinning) and (ii) into a lightly bindered dry fibre preform (tufting).

After the initial insertion of the microfasteners, the Z-pinned prepreg stack or component is cured between rigid tooling faces under elevated pressure and temperature, usually in an autoclave. In the case of a tufted preform, this first needs to be removed from the supporting foam base, which has served to grip the tuft loops on the underside of the part. It is then placed in the final mould and injected with suitable low viscosity resin prior to cure at an elevated temperature.

In practice, the insertion of the microfasteners can be controlled well, both in terms of insertion angle and insertion depth. However, subsequent small movements and deformations in the consolidation and cure stages of the processing, while the matrix resin retains some fluidity, are much more difficult to manage [6]. The effects of microfastener misalignment on the desired out-of-plane mechanical performance enhancement are considerable and the possibility of mis-alignment, therefore, causes a level of uncertainty in the performance of a *z*-direction-reinforced structure. Further progress, therefore, needs to be made in the control of the processing steps and in QA of these three-dimensional structures. In the meantime, it is possible to evaluate the effects of the structural parameters in a series of carefully designed and controlled experiments, which lead to the creation of analytical and/or numerical models over a range of scales. This paper presents the testing and characterization methodology and indicates how our understanding of the effects of the different structural parameters on the micromechanisms of failure under different loading conditions informs the model development strategy.

## 2. Single microfastener tests and models

**Figure 2.**
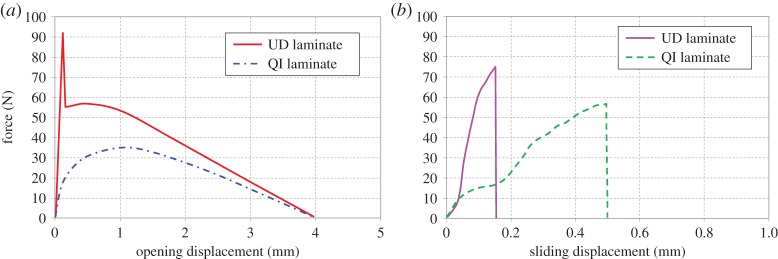
Schematic curves of (*a*) Z-pin mode I pull-out force–displacement and (*b*) Z-pin mode II shear force–displacement. (Online version in colour.)

The most fundamental test to carry out involves a solid square section block of the appropriate composite material which has had a single microfastener inserted vertically into it and a thin polymeric release film placed in the mid-section. The block can then be tested in tension, establishing the force required to pull the microfastener out from its environment, or it can be loaded at various proportions of loading modes up to pure shear [7]. The presence of the mid-plane polymeric film eliminates any loading contribution that would otherwise have come from deformation and fracture of the composite bed. The loads involved in these tests are understandably small and sensitive load cells and digital strain mapping are required for sufficiently accurate measurements. In the case of rigid slender Z-pins, which are manufactured by pultruding aligned carbon fibre tows impregnated with a high temperature thermosetting resin, their lengths tend to be below the critical length for rupture and hence they pull out from the composite if simple tensile loading is applied. The corresponding load–displacement plot (figure 2*a*) exhibits the expected features of elastic stretching followed by de-bonding and pull-out and constitutes the basic crack bridging law (in crack opening mode) that has to be matched by any analytical or numerical models aiming to describe the behaviour of the microfastener in the given composite foundation and composite thickness. The friction between the Z-pin surface and its immediate surroundings is the determining parameter of the crack opening bridging law. As figure 2*a* also shows, the de-bonding of the Z-pin from its surroundings depends on the laminate lay-up and on the level of constraint and residual stress. During laminate manufacture the Z-pin contracts in the transverse direction as the laminate cools from the cure temperature. The laminate plies also contract thus setting up residual stresses between the pin and the surrounding laminate, which can be high enough to initiate a disbond [8]. In the case of the UD material, the laminate contraction is anisotropic, and hence the residual stresses are lower, thus resulting in a higher initial peak load as the pin–laminate interface first fractures. In the case of the multi-directional laminate, the fibres in the different directions provide additional constraint in the lateral direction and so the residual stresses are higher, reducing (or even eliminating) the initial high peak load seen in the UD case and also reducing the overall peak load achieved during pull-out.

Under pure shear-mode loading a different behaviour is observed, in which the fibrous pin splits in the longitudinal direction, starts to deform locally and ultimately ruptures catastrophically (figure 2*b*). It is worth noting that the complex deformation of the Z-pin under shear introduces a small proportion of opening displacement in the test, superimposed onto the main sliding displacement. It is necessary to constrain the test piece against this mode I component if response to pure shear loading is to be recorded. Intentional mixed mode loading results in combinations of the opening mode frictional pull-out and of the shear mode rupture, with a transition between the two behaviours. As with the mode I force–displacement curves, some differences can be observed between curves for different foundation laminates, due to the local conditions at the Z-pin–laminate interface.

**Figure 3.**
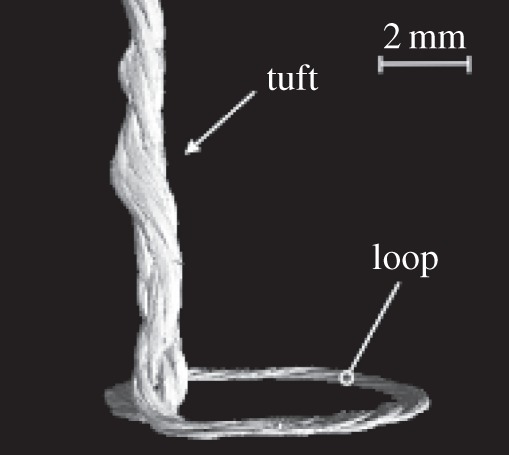
CT scan of a tuft formed from a glass thread within carbon fibre fabric/epoxy composite. The tuft loop has been flattened against the rigid tooling face in the course of resin infusion and cure of the part. Image courtesy: Ms Osmiani.

**Figure 4.**
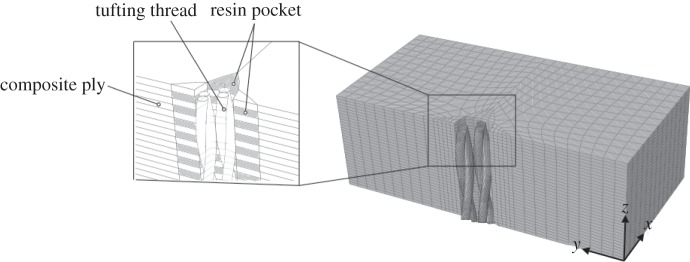
FE model representation of an idealized tuft segment in a composite [9].

In comparison with Z-pins, resin-impregnated tufts have a much more complex internal structure, as the CT scan in figure 3 indicates. The degree of twist, the number of twisted strands as well as the actual thread material (glass, carbon, aramid or metal) will determine the relationship between the axial stiffness and tensile strength of the solid tuft, in turn, determining how a tuft stretches and breaks under given conditions. A high-fidelity finite-elements (FE) model, constructed as indicated in figure 4, is being developed that accounts for such microstructural and/or material variables in predicting the failure of the tuft in a single-tuft test [9].

A major difference between Z-pins and tufts is the presence of the top seam stitches in tufted preforms, which anchor a tuft, preventing pull-out and hence limiting the otherwise beneficial effect of the intrinsically higher surface roughness of the impregnated tuft.

## 3. Model development strategy

**Figure 5.**
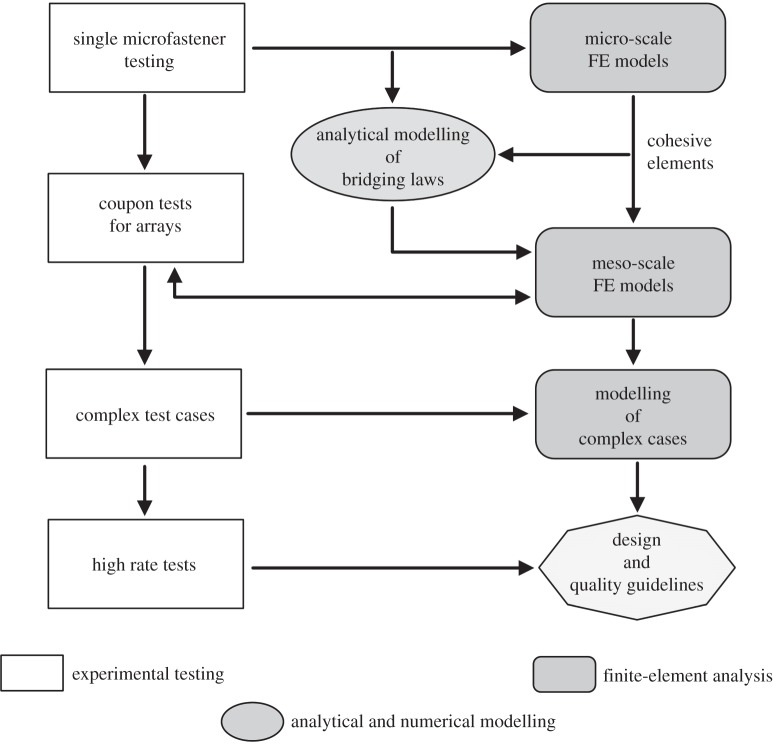
Schematic of the modelling strategy for delamination performance of through-thickness reinforced composites.

A simplified road map for the development of the modelling tools required to capture the out-of-plane load response of through-thickness reinforced composites is shown in figure 5. The left-hand side indicates the minimum necessary experimental programmes that need to be carried out to establish the correct failure mechanisms and to quantify the parameters required in each corresponding stage of the analytical or numerical modelling. The authors’ group has put a lot of emphasis on the single microfastener tests, as the basis of model development. Much of the earlier reported experimental work has tended to concentrate on the coupon array tests, i.e. the commonly used interlaminar fracture testing using double cantilever beam tests, modified to ensure that the test determines the resistance to a running crack, elucidating the development of the bridging zone [1,2,10–13]. It continues to be used as a suitable coupon test for more detailed probing of effects of structural parameters such as, for example, the aspect ratio of the embedded microfastener [14]. As regards more complex loading cases, the most commonly used test element has been the T-joint [15,16]. As the currently developing modelling tools mature, it will be opportune to manufacture and test more complex reinforced structures, as validation of the predictive modelling. Test procedures are now being developed to enable detailed observation of failure mechanisms during high rate delamination and to quantify any rate effects. For all these experimental tests, information on reproducibility of test data are being collated in individual laboratories; a more joined approach is probably required to arrive at systematic development of a sufficiently wide database.

Turning to the right-hand side of figure 5, the modelling stream, its earliest components were the generic analytical models for single tow, stitch or rod bridging, developed by Cox and co-workers [17] and a number of finite-element models for the mode I delamination fracture tests [18,19]. Cohesive zone models (CZM) embedded in special purpose finite elements known as interface or cohesive elements have become popular in recent years for modelling interfacial failure such as composite delaminations [20]. These are based on a stress-based initiation criterion and an energy-based propagation criterion, and are generally used to model the fracture and crack propagation at the resin-rich interfaces of unreinforced composites. A number of researchers sought ways of applying this approach to characterizing the delamination behaviour of through-thickness reinforced composites, validating their models on the limited experimental database available at the time [21]. The authors’ team has made use of its extensive and self-consistent experimental database to create a new consolidated modelling pathway, outlined in the following section.

## 4. Consolidated models: microscale to mesoscale

The effects of mode mixity, caused by deliberate loading or by inclination of the microfastener, have been captured in a new semi-analytical model, which incurs a relatively low computational cost and has been validated for the case of individual Z-pins against a detailed experimental database [7,8]. This model treats the Z-pins as Euler–Bernoulli beams embedded in an elastic foundation. There are two main energy dissipation components considered in the analysis; the work done by frictional forces against pull-out of the pin, and energy expended in internal shearing and eventual fracture of the pin as a result of high shear displacements. By considering the equilibrium of forces that relate to the delamination opening/sliding and the bridging of these displacements by the Z-pins, expressions for the traction–displacement relationships can be derived. The output of the analysis is a continuous ‘bridging map’, indicating the loci of the bridging forces expressed as functions of all the possible crack opening and shear displacements. These analytical models are also able to account for variations in quality, such as insertion angle.

To better understand the detailed behaviour at the single microfastener level, highly detailed finite-element models can be created at this scale for tufts (figure 4) and Z-pins [22], modelling such detail as the resin-rich pocket surrounding the pin, pin misalignment and splitting within the pin. Experimental tests at this scale are time consuming and require diligent care and attention to detail, but still only yield information that can be gleaned from external observations. Creating high-fidelity models of these same test configurations gives a virtual testing capability that can be used for deeper investigation of the internal failure mechanics and also wider parametric studies than can be achieved in a single microfastener experimental testing programme.

**Figure 6.**
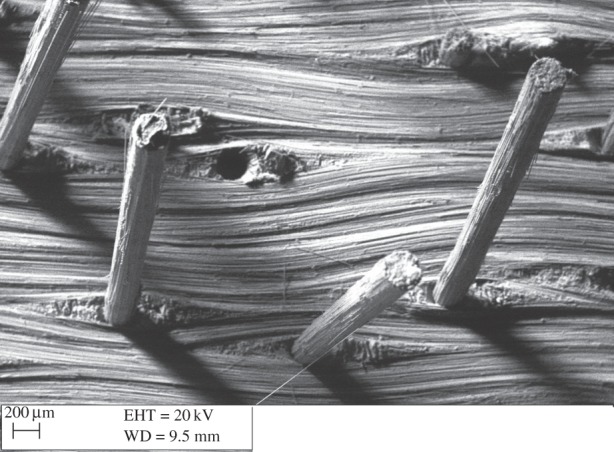
Scanning electron micrograph of carbon fibre/BMI Z-pins pulled out from a quasi-isotropic lay-up carbon fibre/epoxy laminate during a crack opening delamination test of a double cantilever-beam specimen. Resin-rich eyelet features and tow deviation are apparent on the fracture surface.

The deformation and failure of the microfasteners becomes potentially even more complex once they are arranged and inserted in regular arrays, as the possibility of interactions between them cannot be ignored. Resin-rich pockets or ‘eyelets’ are a feature of any planar laminate disturbed by the insertion of an orthogonal microfastener; their shape and size is defined by the detail of the ply direction sequence. The tows which border the resin-rich pockets exhibit significant waviness (figure 6). On this length scale, the micromechanics of damage accumulation under loading becomes a complex interplay between these induced additional structural features and other forms of defects which would have existed in an unreinforced composite such as voids, ply waviness and fibre breakage. No models have been developed to date to account for such complexity. In any case, the computational intensity required by the type of a microstructural model illustrated in figure 4 cannot be sustained into modelling at the level of structural coupons; CZM become an attractive solution to the problem.

Using the bridging maps developed from the analytical bridging laws [8], it is possible to create a new formulation of cohesive element behaviour that is a superposition of the unreinforced interface traction–displacement behaviour and the Z-pin traction–displacement law, smeared over the element surface area. The continuous nature of the analytical law over the displacement space means that just one set of parameters, experimentally derived from single pin tests, can be used for any general loading case. This has been implemented via user subroutine in the commercial finite-element solver Abaqus/Explicit [23], and shown to give good results when compared with experimental data across a range of Z-pin array tests at different mode mixities; double cantilever beam (DCB), end loaded split (ELS) and mixed mode bending (MMB). Success of such models means that for the first time a capability exists for the design of any composite structural element with microfastener through-thickness reinforcement, allowing assessment of the interaction between the microscale bridging behaviour of individual microfasteners and the global load-carrying capacity of the laminate coming from external boundary conditions. That is not to say the problem is solved without future challenges; in particular, the fact that microfastener behaviour is strongly dependent on the local conditions of their insertion, such as laminate thickness, orientation and foundation material, means that significant further development is still required to achieve a full structural-scale design capability.

## 5. Modifications of in-plane properties

The reductions in the in-plane strength of the composite, which inevitably accompany the sought after out-of-plane performance enhancement, will need to be considered carefully in future designs [24]. Not doing so risks the appearance of unexpected new failure modes in through-thickness reinforced structures. For example, quasi-static loading tests of Z-pinned or tufted structural test elements have shown them to reach considerably higher maximum loads than their equivalent control structures but with the energy absorption localized in different parts of the structure and often leading to surface fibre fractures or even through section failures [15,16]. Such failure modes may be highly undesirable in the specific context of the use of that particular structure, creating a new problem that may even be more serious than the initial delamination problem being tackled. In other words, it is possible to have too much of a good thing and a suitable balance needs to be achieved and captured in future global structural models.

To date, sufficient experimental work has not yet been done to establish adequate databases on the effects of microfastener inclusion on the resulting quasi-static properties of the composite [25,26]. Unlike in the case of delamination, the effect of a microfastener array will probably be more dependent on the geometry of the array than on the material properties of the actual microfasteners [27]. It will once again be highly dependent on the topology of the fibre bed as it is the perturbation in this structure that determines the eventual property knockdown. Thus, quasi-isotropic arrangement of fibre layers will be affected less than a unidirectional composite. The range of in-plane strength knockdowns observed is between 0% and 40%, depending on the topology and the loading on the specimen. The worst scenario would be a high areal density (approx. 4%) of microfasteners in a unidirectional composite subjected to compressive loading. It is possible for the resin-rich eyelets to become merged together thus failing to provide resistance to buckling of the in-plane fibres and resulting in premature failure of the specimen [28].

Contrary to commonly expressed expectation, there is relatively little breakage of in-plane fibres incurred by the processes of microfastener insertion. In the case of Z-pin insertion, the action of the ultrasonic gun produces sufficient heat to lower significantly the viscosity of the uncured resin matrix, thus allowing the fibre tows to move around the intruding Z-pin. Similarly, thread-less passage of the tufting needle though a dry fibre preform has been shown to have very little effect on the tensile stiffness and strength of the final infused and cured laminate, compared with control. It is the locking effect of the crimp in the in-plane fibres introduced by the tuft seam that initiates the damage under loading (JWG Treiber 2012, unpublished data).

## 6. Fatigue, low energy impact and high rate loading

Even less work has been carried out on examining the consequences of through-thickness reinforcement on resistance to fatigue loading. Regarding resistance to delamination crack growth, the result is positive, with fatigue crack growth generally inhibited by the presence of Z-pins [29]. In-plane tensile fatigue in Z-pinned laminates has been examined by Mouritz & Chang [30], with no particular concerns on performance being raised. Some of the first work to look at the fatigue performance of a single Z-pin, in which both the wear-out of the pin and the pin–laminate interface were investigated, has recently been reported [31]. Following a similar modelling strategy described above, as employed for the quasi-static loading case, this information will eventually be used to inform model development and application to larger structural fatigue loading cases.

Delamination is commonly the result of transverse impact loading to composite structures. Such impacts can take place at low velocity, e.g. tool drop, leading to barely visible impact damage (BVID), or at much higher loading rates such as bird strike. As a measure to resist delamination propagation from such events microfastener reinforcement needs to be able to perform at a range of loading rates. Some work has been done to show the effectiveness of microfasteners in resisting delamination because of drop weight impact and the resulting compression after impact (CAI) strength improvement, compared with the same test on an equivalent control specimen [4,32]. Such studies have demonstrated significant reductions in the average damage area caused by a falling dart at impact energies of around 20 J. It is possible to tailor the extent of the damaged region by selected placement of microfastener arrays around the impact point and the modelling tools described here should help with optimization of array placement in this context.

To date, there has been very little work to quantify the effect that loading rate has on microfastener reinforcement, from a fundamental understanding point of view. As part of a multi-scale modelling strategy, this is an essential ingredient so as to avoid having to characterize each specific loading scenario empirically.

## 7. Discussion

The most likely threat that through-thickness reinforcement is intended to counter is a potentially high-rate impact on an unknown part of an engineering structure. Such events could be a bird strike or runway debris strike in aircraft context or side impact on structures such as car bodies, rail structures or marine vessels. Commercial use of microfasteners to date has been largely limited to successful but costly post-design fixes of delamination problems in high-value structures. The generic technology is now under evaluation for wider use in a number of specifically designed structures, where a delamination issue is to be avoided. In this context, the tufting technology adoption is likely to benefit from lessons learnt previously from two-sided stitching and Z-pinning. It is aided to a large extent by the progress in multi-scale modelling and also by the association with the lower processing costs of dry fibre preforms and liquid resin infusion. Recently, thread manufacturers have been increasing the range of quality threads suitable for tufting, including multi-material threads, which offer the possibility of introducing new levels of multi-functionality into composite structures.

The above-described modelling strategies are currently able to predict delamination failure and its mitigation across a range of length scales and complexities. Future work will continue to develop the multi-scale strategy, to achieve less computing-intensive local–global models, enabling full scale structural design. The present capability can already account for the effects of known defects in reinforcement patterns, such as microfastener misalignment, incomplete or missing insertion and for known changes in fibre bed thickness and topology. There remains a challenge to model the as-manufactured condition rather than just a range of possibilities. However, it is not feasible to employ very detailed and costly non-destructive inspection procedures to any other than the most critical of structures. It is therefore required to both improve quality control and so reduce anomalies and also statistically characterize the actual variances so that they can be embedded into the models.

The use of through-thickness reinforcement has so far proved to be beneficial in preventing or reducing localized delamination growth under conditions of quasi-static loadings, under low rate impact and under some fatigue conditions. For bespoke design of highly stressed engineering structures the eventual models need to account for the probable presence of multiple delaminations and for the in-plane property reductions which could cause an undesirable change in the dominant failure mode of the structure. The modelling methods also need to be adapted to the cases of very high rate loadings, for which new detailed experimental datasets are required. We can never know where exactly on the structure an impact event will happen and hence where the localized reinforcement would be most effective. However, it can be determined which parts of the structure must not be allowed to delaminate, to preserve their critical structural integrity—such locations are a good candidate for preventative placement of microfasteners. Ultimately, if through-thickness reinforcement is to achieve the potential it offers to create lightweight damage tolerant structures, future structural design practices need to evolve beyond the current ‘no growth’ practices [33].

## Data accessibility

No new data were created during this study.
